# Chemical Composition and Lipid Bioactive Components of *Centaurea thracica* Dwelling in Bulgaria

**DOI:** 10.3390/molecules29143282

**Published:** 2024-07-11

**Authors:** Olga Teneva, Zhana Petkova, Ginka Antova, Maria Angelova-Romova, Plamen Stoyanov, Krasimir Todorov, Tsvetelina Mladenova, Tzenka Radoukova, Rumen Mladenov, Venelin Petkov, Anelia Bivolarska, Donika Gyuzeleva

**Affiliations:** 1 Department of Chemical Technology, Faculty of Chemistry, University of Plovdiv “Paisii Hilendarski”, 24 Tzar Asen Str., 4000 Plovdiv, Bulgaria; olga@uni-plovdiv.bg (O.T.); zhanapetkova@uni-plovdiv.bg (Z.P.); maioan@uni-plovdiv.bg (M.A.-R.); 2 Department of Botany and Biological Education, Faculty of Biology, University of Plovdiv “Paisii Hilendarski”, 24 Tzar Asen Str., 4000 Plovdiv, Bulgaria or plamen.stoyanov@mu-plovdiv.bg (P.S.); ktodorov@uni-plovdiv.bg (K.T.); cmladenova@uni-plovdiv.bg (T.M.); radoukova@uni-plovdiv.bg (T.R.); or rumen.mladenov@mu-plovdiv.bg (R.M.); dgyuzeleva@uni-plovdiv.bg (D.G.); 3 Department of Bioorganic Chemistry, Faculty of Pharmacy, Medical University of Plovdiv, Vasil Aprilov Str. 15A, 4002 Plovdiv, Bulgaria; 4 Bristol Myers Squibb, Cruiserath Dr, Cruiserath, Mulhuddart, Co., Dublin 15, Ireland; 5 Department of Medical Biochemistry, Faculty of Pharmacy, Medical University of Plovdiv, Vasil Aprilov Str. 15A, 4002 Plovdiv, Bulgaria; anelia.bivolarska@mu-plovdiv.bg

**Keywords:** *Centaurea thracica*, chemical composition, lipid composition, fatty acids, bioactive components, lipid indices

## Abstract

*Centaurea thracica (Janka) Hayek* is a plant common in southern Bulgaria. The inflorescences were collected during June and September 2021, while their seeds were obtained in September 2021. The chemical and lipid composition of the inflorescences during the vegetation process of the plant were established. A significant decrease in total proteins (from 8.7 to 7.4%), glyceride oils (2.0–1.7%), and ash (4.5–4.2%) content was observed, while the amount of carbohydrates (72.3–77.2%) and fibers (28.7–35.8%) increased. During the vegetation of the plant, the content of oleic and linoleic acids increased up to 2–3 times, while the level of palmitic acid decreased. The lipids from the seeds were rich in oleic (53.0%) and palmitic (36.2%) acids. The tocopherol content in the oils of the inflorescences during vegetation increased from 58 to 110 mg/kg, and the content in the oil from the seeds was 260 mg/kg. The phospholipid content decreased during vegetation, and differences were observed in the composition between the inflorescences and the seeds. The high content of oleic acid, linoleic acid, tocopherols, and phospholipids determine the nutritional and biological value of the oils isolated from *Centaurea thracica*, and contribute to their potential use in various directions.

## 1. Introduction

The genus *Centaurea* includes a large number of species (more than 700) belonging to the Asteraceae family [1]. Members of the genus are only found north of the equator, primarily in the Eastern Hemisphere, the Middle East, and surrounding regions. A particularly rich diversity of these species is observed mainly in Eastern Anatolia and the South Caucasus, followed by the Mediterranean region and the Balkan Peninsula [2,3]. In the Bulgarian flora, the genus *Centaurea* is represented by more than 75 species and is the richest in endemics [4,5]. It consists of diverse representatives with different growth cycles (annual, biennial, or perennial plants). Some of them are traditionally used for raw consumption or as medicinal plants due to their bioactive properties [6,7,8,9,10].

The genus *Centaurea* is one of the most important of the Asteraceae family [11]. The main species are widely used by people to treat various diseases [12]. Studies conducted with extracts of various *Centaurea* species have shown that they have various vital activities such as enzyme inhibitory activity and anti-hemorrhoidal, anti-cancer, and anti-diabetic effects. They also have anti-inflammatory, analgesic, antioxidant, antimicrobial, and antimutagenic properties [13,14,15,16,17,18,19,20].

Plants of the genus *Centaurea* are pharmaceutically valuable due to their secondary metabolites such as phenols and flavonoids, which have positive effects on human health. These substances are responsible for many biological activities, including the overall effect of purifying the body [17,21,22,23,24]. According to Sokovic et al. [25] and Tešević et al. [26], the biological activity is mostly associated with the presence of flavonoids (apigenin, isokaempferide, hispidulin, eupatorin, cirsimaritin, santoflavone, and salvigenin) as well as sesquiterpene lactones (germacranolides, eudesmanolides, elemanolides, and guaianolides). Cnicin is the major sesquiterpene lactone for many *Centaurea* species and it is responsible for cytotoxic activities.

The lipids contain mainly acylglycerols, which are accompanied by minor amounts of phosphatides, sterols, fat-soluble vitamins, waxes, hydrocarbons, etc. The type and amount of fatty acids in acylglycerols, as well as the position and distribution of these fatty acids in them, determine the chemical, physical, and functional properties of lipids. Clinical and epidemiological studies show that many chronic diseases, such as cardiovascular, cancer, and degenerative diseases, are associated with increased consumption of saturated and trans fatty acids, while intake of monounsaturated and polyunsaturated fatty acids is associated with better health [27,28,29,30,31,32]. Plant lipids contain more long-chain unsaturated fatty acids in their molecule, which easily undergo oxidative changes under the action of atmospheric oxygen. Tocopherols belong to the microcomponents of lipids, and are one of the most important biologically active substances in them. In plants, α-tocopherol is biosynthesized via β- or γ-tocopherol [33]. Tocopherols are one of the most important antioxidants and lipid stabilizers against oxidation. They exert their antioxidant effect through multiple biochemical and biophysical mechanisms, including the deactivation of active free radicals. α-Tocopherol has a stronger antioxidant effect than γ-tocopherol, but it can also act as a “pro-oxidant” under certain conditions [34]. The lipids are an important nutrient, a major source of energy, and transport fat-soluble vitamins.

The fatty acid composition can serve for evaluation of the quality and nutritional properties of the oil. The fatty acid composition of aerial parts from six species of *Centaurea*—*C. balsamita*, *C. calolepis*, *C. carduiformis* subsp. *carduiformis*, *C. cariensis* subsp. *maculiceps*, *C. cariensis* subsp. *microlepis*, and *C. iberica*—was studied. The main fatty acid identified in the oils of all species was linoleic acid (C18:2), except *C. cariensis* subsp. *microlepis*. Other predominant fatty acids in the oils isolated from *Centaurea* species are palmitic (C16:0), linolenic (C18:3), oleic (C18:1), and stearic (C18:0) acids [35]. The significant amounts of linoleic and linolenic acids found in oils isolated from plants of the genus *Centaurea* make *Centaurea* oil a valuable food, i.e., it can be a good source of essential fatty acids that are extremely important in the formation of cell membranes, especially of nervous tissue.

According to some authors, palmitic acid is the main predominant saturated fatty acid in the lipids isolated from *C. salicifolia* subsp. *abbreviata* and *C. babylonica* (from 29.37 to 30.49%) [36]. The data are in agreement with Yildirim et al. [37], who also reported that it is the major fatty acid present in some oils from plants in the genus *Centaurea*.

In recent years, there has been a growing interest in new, previously unexplored plant oils that could be a source of valuable biologically active substances. All this created interest to investigate the chemical composition of the inflorescences of *Centaurea thracica*, as well as the lipid composition of acylglycerols from its inflorescences and seeds, in order to evaluate the possibility of its use as a new alternative source of different biologically active components.

## 2. Results and Discussion

### 2.1. Chemical Composition of Centaurea thracica Inflorescences

The changes in the main indicators determining the chemical composition of the inflorescences of *Centaurea thracica*—content of protein, oil, carbohydrates, moisture, mineral substances, and crude fiber—during the vegetation process of the plant (inflorescences collected in June and September) were established.

The data on the content of the main components in the composition of the inflorescences collected in June and September from *Centaurea thracica* are presented in Table 1.

The inflorescences collected in June from *Centaurea thracica* have a higher protein content (8.7%) and oil content (2.0%) compared to those from September (7.4% and 1.7%). The obtained results for protein content correlate with those of some medicinal and edible plants, for which proteins range from 1.30 to 11.56% [38,39]. A lower carbohydrate content was found in the inflorescences in June (about 72.3%), which increased with the development of the plant by about 5.0%. A higher fiber content is observed in the inflorescences from the month of September (35.8%) compared to their content in those from the month of June (28.7%). The fiber content is close to the results obtained by Vishwakarma and Dubey, who studied various medicinal and aromatic plants and found that their fiber content varied from 0.90 to 28.59% [40]. The inflorescences of *Centaurea thracica* contain mineral salts, and their amount decreases in the process of vegetation of the flower from 4.5 to 4.2%. The content of mineral substances in the inflorescence from the month of September is lower than the data for ash of seeds of the species *Centaurea karduchorum Boiss* (5.93%) [41]. A trend in decreasing moisture content from 12.5 to 9.5% was observed in the last investigated inflorescence.

Generally, the aerial parts of the plants at the beginning of their development mainly consist of water, proteins, and carbohydrates. In the process of vegetation, a decrease in the amount of water and accumulation of the dry matter is observed, as well as the favored formation of reserve non-nitrogen substances, such as sugars, starch, and cellulose. In this regard, the highest increase was observed in the amounts of the carbohydrates, including fibers in the inflorescences from the examined plants.

The increase in carbohydrate content in plants during vegetation is primarily due to photosynthesis. During this period, the plant actively synthesizes, uses, and stores carbohydrates to support its development and ensure survival through less favorable conditions. The decrease in protein content in the plants during vegetation is primarily due to the redistribution of nitrogen to support the growth of plants’ parts other than the inflorescence.

The optimal ratio of the carbohydrates and proteins in plants for edible purposes varies depending on the dietary needs. A balanced diet typically includes a mix of foods with varying carbohydrate-to-protein ratios. For overall dietary balance, aiming for an average ratio in the range of 4:1 to 7:1 might be suitable, but this can vary based on individual nutritional needs. This ratio in the inflorescence in June was 8:1, and in those from September was 10:1.

Data on the chemical composition of the inflorescences of *Centaurea thracica* studied by us are close to the literature data on protein content (10.93%), ash (5.93%), and fiber (32.58%) in the plant *Centaurea karduchorum Boiss* grown in Turkey [41].

The results obtained by us are higher than the literature data for protein content (1.60–2.89%), lipids (0.80–1.39%), mineral substances (1.20–1.56%), and of fiber (14.8–17.2%) when studying the bud of the genus *Centaurea* during three stages of its development (tight bud, mature bud, and fully open) [42].

The inflorescences in September of *Centaurea thracica* have a higher content of carbohydrates, which determines, at a great extent, the increase in the energy value compared to that of the inflorescences in June (from 1453 kJ/100 g (342 kcal/100 g) to 1503 kJ/100 g (354 kcal/100 g)). The energy value of *Centaurea thracica* inflorescences is close to that of herbs or medicinal plants, which have an average energy value of 314 kcal/100 g. The energy value of the inflorescences in June is close to the literature data on the energy value of lentils and potatoes (1449 and 1463 kJ/100 g), while this value of inflorescences in September is similar to that of corn (1577 kJ/100 g) [43].

### 2.2. Lipid Composition of Oil Isolated from Inflorescences and Seeds of Centaurea thracica

The lipid composition of the glyceride oils isolated from the inflorescences and seeds of *Centaurea thracica* was investigated, i.e., fatty acid composition of the oils, tocopherol content, and the content and composition of the phospholipid fraction.

#### 2.2.1. Fatty Acid Composition

Table 2 presents the fatty acid composition of acylglycerols isolated from inflorescences and seeds of *Centaurea thracica*.

The composition of the oil from the inflorescences of *Centaurea thracica* contains 23 fatty acids, and the oil from seeds contains 20 fatty acids. Palmitic acid (32.6%) predominates in the composition of the oil from the inflorescences collected in June, followed by linoleic (13.8%) and oleic acid (13.7%), which are in comparable quantities. There is a relatively high content of lower saturated fatty acids—butyric (C4:0—3.4%) and caproic (C6:0—3.1%)—as well as higher saturated fatty acids—stearic acid (C18:0–4.9%), arachidic (C20:0—3.5%), behenic (C22:0—2.9%), and lignoceric (C24:0—2.8%). The oil also contains a larger amount of the essential linolenic acid (C18:3—3.6%) compared to traditional sunflower oil (0–0.3%), but its amount is close to that in soybean oil (4.5–11.0%) [44].

In the composition of the oil from the inflorescences collected in September, oleic acid (C18:1—30.9%) predominates, followed by palmitic acid (C16:0—21.6%) and linoleic acid (C18:2—20.3%). During the vegetation process, a significant increase in the amount of oleic (from 13.7 to 30.9%) and linoleic (from 13.8 to 20.3%) acids was observed. A threefold increase in the content of palmitoleic acid (C16:1—from 3.8 to 11.7%) and a decrease in the amount of the saturated fatty acids were found, as follows: arachidic acid (C20:0—from 3.5 to 0.2%), behenic (C22:0—from 2.9 to 1.5%), and lignoceric (C24:0—from 2.8 to 1.0%), as well as linolenic acid (C18:3—from 3.6 to 0.7%).

The glyceride oil from the seeds of *Centaurea thracica* is rich in oleic (53.0%) and palmitic (36.2%) acids. Linoleic and linolenic acids are represented in insignificant amounts, 1.4% and 1.1%, respectively. The presence of the essential docosahexaenoic acid in the amount of 1.0% is also noticeable in the seed oil. The content of stearic acid is 3.7%, and the remaining fatty acids are represented in insignificant amounts—from 0.1 to 0.7%. The fatty acid composition of *Centaurea thracica* seed oil is close to that of palm–oleic oil, in which palmitic (38.0–43.5%) and oleic (39.9–46.0%) acids predominate [44].

In the study of seeds from various species of plants of the genus *Centaurea*, different data on the fatty acid composition were reported. The obtained results are different from the data by other authors regarding the composition of oils from different species of plants of the genus *Centaurea* (*Centaurea albonitens* and *C. balsamita*), in which the main acids are linoleic (49.94–47.75%), oleic (30.36–30.07%), palmitic (8.98–10.64%), and stearic (5.89–6.41%) acids [45]. In the literature, there are data on the fatty acid composition of glyceride oils isolated from the bud of the genus *Centaurea* during three stages of its development (tight bud, mature bud, and fully open). It was established that the main fatty acids were oleic (from 9.04 to 26.4%), palmitic (from 24.8 to 30.0%), and linoleic (from 12.8 to 20.7%) acids [42]. This composition is close to the fatty acid composition of the oil isolated from the inflorescences collected in September of *Centaurea thracica*.

The analyzed oils were rich of palmitic acid and our results are close to the reported by Aktumsek et al. [36], who found that palmitic acid was the main one in the oils isolated from *C. salicifolia* subsp. *abbreviata* and *C. babylonica* (from 29.37 to 30.49%). The results are in agreement with Yildirim et al. [37], who reported that the palmitic acid was also the major one in some oils from plants of the genus *Centaurea*.

There are some differences in the fatty acid composition of the oils obtained from the three investigated *Centaurea* species (*C. patula*, *C. pulchella*, and *C. tchihatcheffii*), where the content of linolenic (C18:3—24.01–33.62%), linoleic (C18:2—18.92–34.55%), palmitic (C16:0—13.22–21.02%), and oleic (C18:1—1.44–11.30%) acids were established [46]. The presence of the essential docosahexaenoic acid was also found in an amount of 0.56% to 0.97%, which is similar to its content in the oils of the unripe inflorescences and the seeds of *Centaurea thracica* in the present study.

The fatty acid compositions of six species of *Centaurea*—*C. balsamita*, *C. calolepis*, *C. carduiformis* subsp. *carduiformis*, *C. cariensis* subsp. *maculiceps*, *C. cariensis* subsp. *microlepis*, and *C. iberica*—were also determined. According to Tekeli et al. [35], the major fatty acid identified in all species was linoleic acid (C18:2—31.23–40.60%), except for *C. cariensis* subsp. *microlepis* (23.92%). Other predominant fatty acids in *Centaurea* oil are palmitic (C16:0—17.83–25.31%), linolenic (C18:3—8.54–27.36%), oleic (C18:1—8.65–27.22%), and stearic (C18:0—2.93–7.18%) acids. The *Centaurea* oil can be a good source of essential fatty acids, and the high content of linoleic acid, which is the main fatty acid of the polyunsaturated fatty acids, makes it a valuable food.

The n-6/n-3 ratio was found to vary widely from 1.14 in the seed oil to 26.6 in the oil isolated from the inflorescences collected in September. It can be seen that in the oil isolated from the seeds, the amounts of n-3 and n-6 are almost the same, while in the oils isolated from the inflorescences, the content of n-6 significantly exceeds that of n-3. The established n-6/n-3 ratio of the seed oil is close to that obtained by a previous study on the fatty acid composition of glyceride oils isolated from the bud of the genus *Centaurea* at three stages of its development (n-6/n-3 is 0.65–1.00) [42]. The higher ratio of n-6/n-3 in the oil from the inflorescences collected in September is due to the high content of linoleic acid (20.3%) and the insignificant amount of linolenic acid (0.7%). These changes can be explained, as follows, by the different biosynthesis’ stages of the fatty acids: saturated fatty acids predominate in the first stage of development of the plants, and after that, the content of the polyunsaturated fatty acids increases. These ratio values indicate that the studied oils have a lower content of n-3 fatty acids, which is a characteristic of the most vegetable oils.

**Figure 1:**
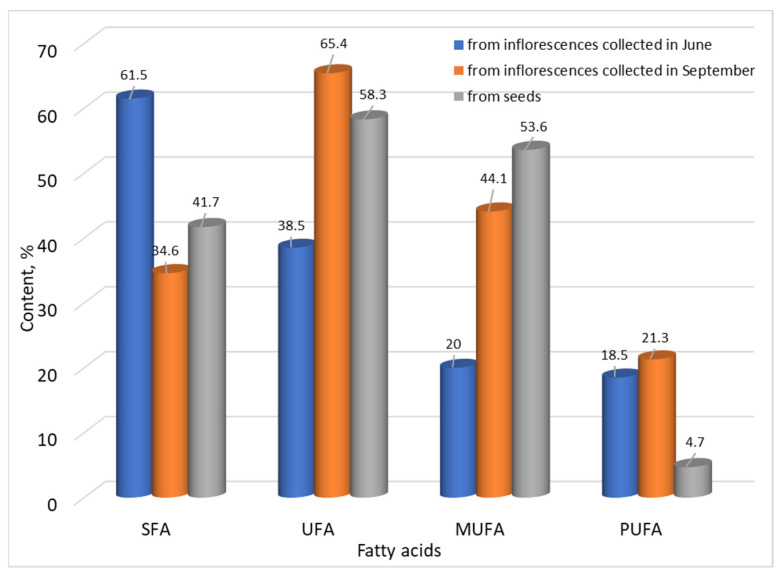
Content of saturated (SFA), unsaturated (UFA), monounsaturated (MUFA), and polyunsaturated (PUFA) fatty acids in oil isolated from inflorescences at different stages of vegetation and seeds of *Centaurea thracica*.

Figure 1 shows the ratio of saturated, unsaturated, monounsaturated, and polyunsaturated fatty acids in the oil isolated from inflorescences at different stages of vegetation and seeds of *Centaurea thracica*.

The amount of saturated fatty acids predominates in the oil isolated from the inflorescences in June (61.5%), while in the oils isolated from inflorescences collected in September and from the seeds, the amount of the unsaturated fatty acids is the highest—65.4% and 58.3%, respectively. The main representative of saturated fatty acids is palmitic acid (21.6–36.2%), followed by stearic acid (3.7–4.9%). The quantities of monounsaturated fatty acids in the oils from inflorescences in September (44.1%) and from the seeds (53.6%) were significantly higher than those of polyunsaturated fatty acids—21.3% and 4.7%, respectively—while in the oil from the inflorescences in the month of June, the ratio of monounsaturated (20.0%) to polyunsaturated (18.5%) fatty acids is almost 1:1. The main representative of monounsaturated fatty acids is oleic acid (13.7–53.0%), while the polyunsaturated ones are linoleic (1.4–20.3%) and linolenic (0.7–3.6%) acids.

The results of our research differ from other studies regarding the fatty acid composition of oils isolated from six *Centaurea* species, where polyunsaturated fatty acids predominate (41.02–58.80%), and the amount of monounsaturated fatty acids varies from 9.43% to 28.56% [35]. The content of saturated fatty acids in the oils from inflorescences and seeds that we studied is close to that obtained by studying the fatty acid composition of glyceride oils isolated from the bud of the genus *Centaurea* during the three stages of its development (SFA—37.0–49.0%), but the amounts of MUFA and PUFA fatty acids differ significantly from our results (MUFA from 10.0 to 27.0%, and PUFA from 32.0 to 42.0%) [42].

The obtained results differ compared with the data about the composition of oils from different species of *Centaurea* plants (*Centaurea albonitens* and *C. balsamita*) reported by Peker and Baştürk [45], in which polyunsaturated fatty acids predominate (50.11% and 47.96%, respectively), monounsaturated are found to be 31.22% for both species, and saturated fatty acids are found to be 17.25% and 18.81%, respectively. These oils, compared to the ones we studied, have a significantly lower content of saturated fatty acids (2–3 times) and a higher content of polyunsaturated fatty acids (from 2 to 10 times).

The iodine value, which is an indicator of the degree of unsaturation of fatty acids in the oils, is relatively low (47.2–66.5 g I_2_/100 g), and this is due to the high content of saturated fatty acids in the studied oils. Their iodine value is close to that of palm–olein oil (≥56 g I_2_/100 g) [44]. For that reason, the investigated oils can be classified as non-drying oils, which are characterized using an iodine value between 50 and 100 g I_2_/100 g.

Based on the data about fatty acid composition of both oils (from seeds and inflorescences) of *Centaurea thracica*, we determined the following indicators which are related to the evaluation of the benefits of the oil for human health and which are the criteria for their therapeutic effect: the ratio between polyunsaturated and saturated fatty acids, and the atherogenic and thrombogenic index.

Table 3 presents the data regarding the ratio between polyunsaturated and saturated fatty acids, and the atherogenic and thrombogenic index.

The ratio of polyunsaturated and saturated fatty acids has an important role in determining the various properties of cell membranes that help maintain normal cell metabolism. The recommended minimum ratio of polyunsaturated and saturated fatty acids is 0.45, and the optimal ratio, which leads to a reduction in the risk of cardiovascular diseases, is 1.0–1.5 [47]. For inflorescence oils, it is 0.30 and 0.61, respectively, which is close to the recommended minimum ratio, but that for seed oil is significantly lower than the optimal value, which is a result of the low content of polyunsaturated fatty acids. The PUFA/SFA ratio of the investigated *Centaurea thracica* inflorescences and seed oils was much lower than the same ratio of soybean oil (4.39), corn oil (4.10), sesame oil (2.94), and was close to that of palm oil (0.18) [47].

The atherogenic index shows the relationship between the sum of the main saturated fatty acids, which are considered to be pro-atherogenic (they cause an increase in the level of cholesterol in the blood because they are easily deposited on the walls of the arteries) and the main unsaturated fatty acids, which have an anti-atherogenic effect [48]. The oil from the inflorescences collected in June has a twice higher atherogenic index (1.20) than the oils isolated from the inflorescences in September and the seeds (0.50 and 0.64). Plant oils are characterized by different values of the atherogenic index: palm oil—0.88; olive oil—0.14; and sunflower oil—0.07. Anti-atherogenic lipids inhibit the accumulation of plaque and reduce the levels of esterified fatty acids and cholesterol, thus preventing the occurrence of coronary diseases [49,50].

The thrombogenic index determines the tendency to induce thrombogenesis in blood vessels. The values of this index for the studied oils ranged from 0.82 to 1.33, while for other plant oils, they were as follows: palm oil—1.74; olive oil—0.32; and sunflower oil—0.28 [50].

Atherogenic and thrombogenic indices lower than 1.0 are considered to be indicative of better antiatherogenic and antithrombogenic properties of lipids [51]. The values of these indices for the oil isolated from the inflorescences in September were lower than 1.0, while those collected in June were slightly over 1.0 but lower than 1.5.

Based on the results, the oil from the inflorescences and seeds of *Centaurea thracica* may eventually find application in food, cosmetic, and pharmaceutical products.

#### 2.2.2. Content of Tocopherols

The results regarding the tocopherol content and individual composition of tocopherol fraction are presented in Table 4.

The highest content of tocopherols was observed in the oil isolated from the seeds—260 mg/kg. The total content of tocopherols in the oil of the inflorescences increases about 2 times during their growing season—110 and 58 mg/kg, respectively. The content of tocopherols in the examined oils is close to that of palm kernel oil (0–260 mg/kg) and that of grape seed oil (240–410 mg/kg), but it is significantly lower than that of traditional sunflower oil (440–1520 mg/kg) [44].

Significantly higher values for the content of tocopherols were obtained by Peker and Baştürk, who examined oils from different species of plants of the genus *Centaurea*: *Centaurea albonitens*—1689 mg/kg, and *C. Balsamita*—1186 mg/kg [45].

The amount of tocopherols in the studied oils is close to that of *Camellia sinensis* seed oil (210 mg/kg) [52] and rapeseed oil (190 mg/kg) [53].

The presence of only one representative of tocopherols (α-isomer) was found in the investigated oils from inflorescences and seeds of *Centaurea thracica*. The tocopherol composition matches that of oils isolated from *Centaurea albonitens* and *C. balsamita*, in which α-tocopherol is also predominant. The individual tocopherol composition of *Centaurea thracica* inflorescences and seed oil is close to the composition of sunflower and safflower oil, which mainly contain α-tocopherol [44].

The results obtained by us differ from those in the study of the tocopherol composition of oils isolated from the bud of the genus *Centaurea* during three stages of its development. Fernandes et al. [42] found that during the inflorescence’s development of plants of the genus *Centaurea*, the tocopherol content in the isolated oil decreased from 3.0 to 2.4 mg/100 g dry weight, and their oils contained all four major representatives of tocopherols (α-, β-, γ-, and δ-tocopherol), with α-tocopherol being predominant.

#### 2.2.3. Phospholipid Content and Individual Phospholipid Composition

The results regarding the total phospholipid content and the individual phospholipid composition of the lipids isolated from the inflorescences and seeds of *Centaurea thracica* are given in Table 5.

The phospholipid content decreased twice during inflorescence development, from 0.70% to 0.35%, while the amount of phospholipids in lipids from the seeds was found to be 0.30%. The higher percentage of phospholipids in the lipids isolated from the inflorescence in June is due to the fact that they are mainly synthesized in the initial stage of vegetation. Their phospholipid content is similar to that in sunflower, linseed, corn, soybean, and rapeseed oils (0.7–1.0%) [54].

In the phospholipid fraction of lipids from the inflorescences and seeds of *Centaurea thracica*, almost all major classes of phospholipids were identified. In the phospholipids isolated from the inflorescences in June, phosphatidic acids (22.1%), and diphosphatidylglycerol (21.4%) predominate. This is followed by phosphatidylinositol (14.6%) and phosphatidylserine (12.5%). The remaining classes of phospholipids are represented in almost the same amount (6–7%), with the exception of lysophosphatidylethanolamine, which is 2.5%. During inflorescence development, there was a sharp increase in the amount of phosphatidylcholine (from 6.6 to 23.9%), and to a lesser extent phosphatidylethanolamine (from 6.9 to 13.9%) and lysophosphatidylcholine (from 6.1 to 9.4%), at the expense of reducing the amount of diphosphatidylglycerol (from 21.4 to 7.3%), phosphatidylserine (from 12.5 to 4.7%), phosphatidic acids (from 22.1 to 19.8%), and of phosphatidylinositol (from 14.6 to 10.4%). The presence of monophosphatidylglycerol (1.0%) was also found in a minimal amount. Lysophosphatidylcholine and lysophosphatidylethanolamine are considered to be breakdown products of other classes of phospholipids. Phosphatidic acids can be considered both as degradation products and as a stage in the biosynthesis of the remaining phospholipids. For this reason, their amount is greater in the lipids isolated from the inflorescence during the maturation period.

The phospholipid composition of lipids isolated from seeds is qualitatively similar to that of *Centaurea thracica* inflorescence from both June and September, but quantitatively different. The main representatives of the phospholipids isolated from the seeds were found to be phosphatidylethanolamine (31.8%) and phosphatidylinositol (25.0%), followed by sphingomyelin (11.3%), phosphatidylcholine (9.1%), and phosphatidylserine (7.0%). The amount of monophosphatidylglycerol and diphosphatidylglycerol was 5.6% and 4.9%, respectively, and lysoforms were 2.8% and 2.5%. No phosphatidic acids were identified in the seed phospholipids. The phospholipid composition of seed lipids was closer to that of most plant lipids, in which phosphatidylcholine, phosphatidylethanolamine, and phosphatidylinositol predominate.

## 3. Materials and Methods

### 3.1. Materials

The inflorescences and seeds of the plant *Centaurea thracica (Janka) Hayek*, common in southern Bulgaria, were investigated. The aerial part was collected during the vegetation period (during flowering and seed forming) in the vicinity of the town of Tsarevo, a floristic region near the sandy shore of the Black Sea (Popski Plazh locality), from a population with a high number of the species. The plant material was collected from natural habitats (meadows) from the coastal area. The climatic conditions of the area in June 2021 were as follows: the average temperature was 20.3 °C, and the average rainfall was 69 mm. The average temperature and the average rainfall of the area in September 2021 were 19.3 °C and 49 mm. The flowering inflorescences from *Centaurea thracica* were collected in June 2021, and the mature inflorescences and seeds were obtained in September 2021. Approximately 500 g of fresh plant material were collected randomly. The inflorescences and seeds were air-dried and kept at room temperature in the dark before they were subjected to analysis after grinding.

### 3.2. Chemical Composition

The lipids were isolated by a Soxhlet extraction with n-hexane, and the oil content was determined [55]. The protein content was determined according to AOAC (2016) after mineralization and distillation of the samples [56]. Total carbohydrates were calculated by the following formula: 100 − (weight in grams [protein + lipids + moisture + ash] in 100 g of dry seeds) [57]. The content of fibers, ash, and moisture were determined according to AOAC (2016) [56]. The energy value (EV) in kJ/100 g (kcal/100 g) was calculated as follows:

EV = % proteins × 17 (4.0) + % carbohydrates × 17 (4.0) + lipids × 38 (9.0).

### 3.3. Lipid Composition

#### 3.3.1. Fatty Acids

The fatty acid composition was determined after transesterification of the oil with methanol (with the presence of sulfuric acid) and the following determination with gas chromatography (GC) [58,59]. The conditions of the GC (Agilent (Santa Clara, CA, USA) 8860) are as follows: 70 °C (1 min), at 6 °C/min to 180 °C (0 min), and at 5 °C/min to 250 °C; split ratio—50:1; temperatures of the injector and detector (flame ionization detector) were 270 °C and 300 °C; column—capillary DBFastFAME (30 m × 0.25 mm × 0.25 μm (film thickness)); and the carrier gas was nitrogen. The identification of FAMEs was performed by the retention time of a standard FAME mix (37 components, Supelco, Bellefonte, PA, USA).

#### 3.3.2. Tocopherols

Total and individual tocopherol composition was determined according to ISO 9936 [60], with slight modifications. Tocopherols were determined on an Merck-Hitachi (Merck, Darmstadt, Germany) high-performance liquid chromatograph instrument with a fluorescent detection (295 nm of excitement and 330 nm of emission) using Nucleosil Si 50–5 column (250 mm × 4 mm). The mobile phase was hexane:dioxane, 96:4 (*v*/*v*), and the flow rate was 1 mL/min. A 2% solution of the oil in hexane was prepared, and 20 μL were injected into the instrument. The tocopherols were identified by comparing the retention times with those of the standards (reference individual pure tocopherols—α-, β-, γ-, and δ-tocopherols with purity ≥ 98% from Merck (Darmstadt, Germany)). Tocopherol content was calculated on the base of the tocopherol peak areas in the sample vs. the tocopherol peak area of a standard tocopherol solution.

#### 3.3.3. Phospholipids

The individual phospholipids were isolated by two-dimensional thin-layer chromatography [61]. The obtained phospholipid spots were scraped, placed in tubes, and mineralized with a mixture of perchloric acid and sulfuric acid, 1:1 (*v*/*v*). After that, a water solution of ammonium molybdate and a reducing reagent were added to the samples, and the tubes were kept for 10 min in a boiling water bath in order for the reaction to take place. The quantification was carried out spectrophotometrically at 700 nm. The phospholipid content in the sample was calculated as a percentage of the phosphorus [62].

#### 3.3.4. Iodine Value

The iodine value (IV) was determined according to the procedure by AOCS (1999), as follows [63]:

IV = (90 × % Oleic acid + 181 × % Linoleic acid + 274 × % Linolenic acid)/ 100, g I_2_/100 g.

#### 3.3.5. Index of Atherogenicity and Thrombogenicity

The index of atherogenicity (IA) and thrombogenicity (IT) were calculated according to the formulae given by Ulbricht and Southgate [50]:

IA = (C12:0 + 4 × C14:0 + C16:0)/(∑MUFA + ∑PUFA)(1)

IT = (C14:0 + C16:0 + C18:0)/[(0.5 × ∑MUFA) + (0.5 × ∑n-6 PUFA) + (3 × ∑n-3 PUFA) + (∑n-3 PUFA/∑n-6 PUFA)](2)

where ∑MUFA—amount of the monounsaturated fatty acids, ∑PUFA—polyunsaturated fatty acids, ∑n-6 PUFA—polyunsaturated fatty acids (n-6), ∑n-3 PUFA—polyunsaturated fatty acids (n-3), C12:0—lauric acid, C14:0—myristic acid, C16:0—palmitic acid, and C18:0—stearic acid.

### 3.4. Statistical Analyses

All the analyses were performed in triplicates (*n* = 3). The results are represented as mean and standard deviation (SD).

## 4. Conclusions

For the first time in Bulgaria, detailed research was conducted on the chemical and lipid composition of inflorescences and seeds from *Centaurea thracica*. The results obtained for the general chemical composition of the inflorescences during vegetation show a notable decrease in total proteins, glyceride oils, and ash content, while the amount of carbohydrates and fibers increased. A higher content of carbohydrates was observed in the plants collected in September, which determined the increase in the energy value of these inflorescences. In the composition of the oil isolated from the inflorescences in June, saturated fatty acids predominated, while in the oils isolated from the inflorescences collected in September and from the seeds, unsaturated fatty acids prevailed. The main representatives of the saturated fatty acids were palmitic and stearic acids, of monounsaturated fatty acids was oleic acid, and of polyunsaturated fatty acids were linoleic and linolenic acids. During the vegetation of the plant, the content of oleic and linoleic acids increased about 2–3 times, while the level of palmitic acid decreased. All examined oils were a rich source of essential fatty acids (oleic, linoleic, and docosahexaenoic acids). The values of the indices of atherogenicity and thrombogenicity described relatively good antiatherogenic and antithrombogenic properties of the studied oils, as well as the values of the ratio PUFA/SFA confirmed their possible nutritional and biological properties. The tocopherol content in the oils of the inflorescences increased, while the phospholipids decreased during vegetation.

The high content of essential fatty acids, tocopherols, and phospholipids define the inflorescences and the seeds of *Centaurea thracica* as a good alternative source of biologically active substances with health benefits. The current study involves preliminary examinations on the abovementioned components in the plant. For that reason, there is a need for determining the biological activity of the plant extracts, in which basis can be made conclusions on their potential use in various areas. Apart from that, a detailed examination on the lipid composition of this plant is also a priority because it can give information on its potential utilization in the development of numerous nutritional supplements and pharmaceutical products for the prevention of some chronic diseases.

## Figures and Tables

**Table 1:** Changes in the total chemical composition of *Centaurea thracica* inflorescences.

Indicators	June	September
Proteins, %	8.7 ± 0.1 *	7.4 ± 0.2
Oil, %	2.0 ± 0.1	1.7 ± 0.1
Carbohydrates, %	72.3 ±0.6	77.2 ± 0.7
Crude fibers, %	28.7 ± 0.4	35.8 ± 0.3
Ash, %	4.5 ± 0.1	4.2 ± 0.1
Moisture, %	12.5 ± 0.1	9.5 ± 0.1
Energy value **, kJ/100 g (kcal/100 g)	1453 (342)	1503 (354)

* The samples were analyzed in triplicate (*n* = 3), and the results were expressed as mean and standard deviation. ** The energy value (EV) in kJ/100 g (kcal/100 g) was calculated as follows: EV = % proteins × 17 (4.0) + % carbohydrates × 17 (4.0) + lipids × 38 (9.0).

**Table 2:** Fatty acid composition of glyceride oils isolated from *Centaurea thracica*.

Fatty Acids %		Inflorescences		Seeds
		June	September	
C 4:0	butyric	3.4 ± 0.2 *	- **	-
C 6:0	caproic	3.1 ± 0.1	0.7 ± 0.2	-
C 8:0	caprylic	-	0.1 ± 0.0	0.2 ± 0.02
C 10:0	capric	-	0.2 ± 0.05	0.1 ± 0.0
C 12:0	lauric	2.9 ± 0.2	1.1 ± 0.1	0.1 ± 0.0
C 14:0	myristic	2.5 ± 0.1	2.5 ± 0.15	0.2 ± 0.05
C 14:1	myristoleic	0.5 ± 0.1	0.8 ± 0.2	0.1 ± 0.0
C 15:0	pentadecanoic	1.0 ± 0.2	0.4 ± 0.1	0.2 ± 0.05
C 15:1	pentadecenoic	0.5 ± 0.1	0.1 ± 0.0	-
C 16:0	palmitic	32.6 ± 0.5	21.6 ± 0.6	36.2 ± 0.2
C 16:1	palmitoleic	3.8 ± 0.1	11.7 ± 0.2	-
C 17:0	heptadecanoic	0.9 ± 0.1	0.5 ± 0.06	0.3 ± 0.05
C 17:1	heptadecenoic	0.8 ± 0.1	0.5 ± 0.1	0.3 ± 0.0
C 18:0	stearic	4.9 ± 0.3	4.5 ± 0.2	3.7 ± 0.4
C 18:1	oleic	13.7 ± 0.5	30.9 ± 0.7	53.0 ± 0.5
C 18:2 (n-6)	linoleic	13.8 ± 0.4	20.3 ± 0.3	1.4 ± 0.2
C 18:3 (n-3)	linolenic	3.6 ± 0.3	0.7 ± 0.1	1.1 ± 0.1
C 20:0	arachidic	3.5 ± 0.5	0.2 ± 0.05	-
C 20:1	gadoleic	0.4 ± 0.1	0.1 ± 0.0	-
C 20:2 (n-6)	eicosadienoic	0.5 ± 0.2	0.2 ± 0.05	0.4 ± 0.1
C 22:0	behenic	2.9 ± 0.3	1.5 ± 0.1	0.5 ± 0.1
C 22:1	erucic	0.3 ± 0.1	-	-
C 22:2 (n-6)	docosadienoic	-	-	0.7 ± 0.1
C 20:5 (n-3)	eicosapentaenoic	-	-	0.1 ± 0.0
C 23:0	tricosanoic	1.0 ± 0.2	0.4 ± 0.1	-
C 24:0	lignoceric	2.8 ± 0.4	1.0 ± 0.2	0.2 ± 0.1
C 24:1	nervonic	-	-	0.2 ± 0.05
C 22:6 (n-3)	docosahexaenoic	0.6 ± 0.1	0.1 ± 0.0	1.0 ± 0.2
Σ n-6		14.3	20.5	2.5
Σ n-3		4.2	0.8	2.2
Ratio of n-6/n-3		3.4	26.6	1.14
Iodine value, g I_2_/100 g		47.2	66.5	53.2

* The samples were analyzed in triplicate (*n* = 3), and the results are expressed as mean and standard deviation. **—not detected, Limit of detection is 0.02%, Limit of Quantitation is 0.05%.

**Table 3:** Ratio of polyunsaturated and saturated fatty acids (PUFA/SFA), and the atherogenic and thrombogenic indices of oils from inflorescences and seeds.

Oils From:	PUFA/SFA	Atherogenic Index	Thrombogenic Index
Inflorescences in June	0.30 ± 0.05 *	1.20 ± 0.2	1.33 ± 0.05
Inflorescences in September	0.62 ± 0.02	0.50 ± 0.1	0.82 ± 0.02
Seeds	0.11 ± 0.01	0.64 ± 0.1	1.13 ± 0.03

* The samples were analyzed in triplicate (*n* = 3), and the results were expressed as mean and standard deviation.

**Table 4:** Tocopherol content and individual tocopherol composition of oils from inflorescences and seeds of *Centaurea thracica*.

Content	Inflorescences		Seeds
	June	September	
Tocopherols, mg/kg	58 ± 5 *	110 ± 5	260 ± 10
Individual tocopherol composition			
α-Tocopherol, % of total tocopherol content	100 ± 0	100 ± 0	100 ± 0

* The samples were analyzed in triplicate (*n* = 3), and the results were expressed as mean and standard deviation. Limit of detection is 15 mg/kg.

**Table 5:** Individual phospholipid composition of lipids isolated from inflorescences and seeds of *Centaurea thracica*.

Phospholipids % (from Total Phospholipids)	Inflorescences		Seeds
	June	September	
Phosphatidylcholine	6.6 ± 0.2 *	23.9 ± 0.5	9.1 ± 0.5
Phosphatidylinositol	14.6 ± 0.3	10.4 ± 0.4	25.0 ± 1.5
Phosphatidylethanolamine	6.9 ± 0.5	13.9 ± 0.4	31.8 ± 1.1
Sphingomyelin	7.3 ± 0.1	7.6 ± 0.5	11.3 ± 1.1
Phosphatidylserine	12.5 ± 0.4	4.7 ± 0.1	7.0 ± 1.0
Lysophosphatidylcholine	6.1 ± 0.1	9.4 ± 0.4	2.8 ± 0.4
Lysophosphatidylethanolamine	2.5 ± 0.3	2.0 ± 0.2	2.5 ± 0.6
Monophosphatidylglycerol	- **	1.0 ± 0.1	5.6 ± 0.5
Diphosphatidylglycerol	21.4 ± 0.4	7.3 ± 0.3	4.9 ± 0.2
Phosphatidic acids	22.1 ± 0.1	19.8 ± 0.4	-
Total phospholipid content in the oil %	0.70 ± 0.10	0.35 ± 0.05	0.30 ± 0.04

* The samples were analyzed in triplicate (*n* = 3), and the results were expressed as mean and standard deviation; **—not detected, Limit of detection is 0.02%, Limit of Quantitation is 0.05%.

## Data Availability

The data presented in this study are available on request from the corresponding author.
